# ApoG2 induces cell cycle arrest of nasopharyngeal carcinoma cells by suppressing the c-Myc signaling pathway

**DOI:** 10.1186/1479-5876-7-74

**Published:** 2009-08-23

**Authors:** Zhe-Yu Hu, Jian Sun, Xiao-Feng Zhu, Dajun Yang, Yi-Xin Zeng

**Affiliations:** 1 State Key Laboratory of Oncology in South China and the Department of Experimental Research, Sun Yat-sen University Cancer Center, Guangzhou, PR China; 2 Ascenta Therapeutics Incorporation, Malvern, Pennsylvania, USA

## Abstract

**Background:**

apogossypolone (ApoG2) is a novel derivate of gossypol. We previously have reported that ApoG2 is a promising compound that kills nasopharyngeal carcinoma (NPC) cells by inhibiting the antiapoptotic function of Bcl-2 proteins. However, some researchers demonstrate that the antiproliferative effect of gossypol on breast cancer cells is mediated by induction of cell cycle arrest. So this study was aimed to investigate the effect of ApoG2 on cell cycle proliferation in NPC cells.

**Results:**

We found that ApoG2 significantly suppressed the expression of c-Myc in NPC cells and induced arrest at the DNA synthesis (S) phase in a large percentage of NPC cells. Immunoblot analysis showed that expression of c-Myc protein was significantly downregulated by ApoG2 and that the expression of c-Myc's downstream molecules cyclin D1 and cyclin E were inhibited whereas p21 was induced. To further identify the cause-effect relationship between the suppression of c-Myc signaling pathway and induction of cell cycle arrest, the expression of c-Myc was interfered by siRNA. The results of cell cycle analysis showed that the downregulation of c-Myc signaling pathway by siRNA interference could cause a significant arrest of NPC cell at S phase of the cell cycle. In CNE-2 xenografts, ApoG2 significantly downregulated the expression of c-Myc and suppressed tumor growth *in vivo*.

**Conclusion:**

Our findings indicated that ApoG2 could potently disturb the proliferation of NPC cells by suppressing c-Myc signaling pathway. This data suggested that the inhibitory effect of ApoG2 on NPC cell cycle proliferation might contribute to its use in anticancer therapy.

## Background

Nasopharyngeal carcinoma (NPC) is an epithelial squamous cell carcinoma endemic in Southeast Asia and parts of Mediterranean and northern Africa [1]. Radiotherapy alone cures more than 90% of cases of stage I NPC; however, patients with advanced disease tend to experience therapy failure. Several groups have shown that the 5-year survival rate for concurrent chemotherapy and radiotherapy is higher than that for radiotherapy alone in patients with advanced disease [2,3]. Currently, cisplatin combined with 5-fluorouracil is the first-line chemotherapeutic regimen for NPC. Although this regimen has manageable toxic effects and has yielded response rates ranging from 65% to 75% [4], an urgent need for inpatient administration of chemotherapy has accelerated the development of newer, more tolerable and potent platinum-based regimens. We previously showed that ApoG2 in particular could potently kill NPC cells and had a synergic effect with cisplatin to induce cell death [5]. In this study, we further investigated the effect of ApoG2 on cell cycle regulator proteins and cell cycle progression.

Gossypol and its derivates reportedly induce apoptosis by inhibiting the antiapoptotic function of the Bcl-2 family of proteins [5,6]. Also, authors have found cell cycle arrest in gossypol-treated cells. Several cell cycle-related molecules are involved in gossypol-induced cell cycle arrest. For example, researchers have reported that gossypol-induced cell death was coupled with upregulation of c-Fos expression and biphasic c-Myc expression in rat spermatocytes [7]. Furthermore, transforming growth factor-β is activated by gossypol in prostate cancer cells, and gossypol upregulates p21 expression and downregulates cyclin D1 and Rb expression in colon cancer cells [8,9]. Modifications of these cell cycle-related molecules result in cancer cell arrest at G0/G1 phase of the cell cycle. However, Chang et al. found that gossypol did not affect cell cycle progression or the p53 or p21/WAF signaling pathway in A549 human alveolar lung cancer cells [10]. Different oncogenic pathways are activated in different types of cancer, and treatment with gossypol may have various biochemical and molecular impacts on different cancers with specific biological behaviors.

NPC is associated with Epstein-Barr virus (EBV) infection and genetic susceptibility. EBV-encoded latent membrane protein 1 (LMP1) is a principal oncogene in cases of NPC; it can activate a number of signaling pathways, including nuclear factor-κb, mitogen-activated protein kinase, and phosphoinositide 3-kinase [11]. Besides the LMP1-induced oncogenic pathways, dysregulation of factors such as p16, cyclin D1, and cyclin E leads to aberrations in the cell cycle in NPC cells. Therefore, NPC has multiple unique abnormalities that are potential targets for novel treatments. In this study, we examined the effect of ApoG2 on cell cycle distribution and the involved signal pathways in NPC cells. The results demonstrated that ApoG2 potently arrested cells at S phase of the cell cycle. We also observed that suppression of the c-Myc signaling pathway was responsible for the ApoG2-induced cell cycle arrest.

## Materials and methods

### Cells, Drugs, and Reagents

Poorly differentiated human NPC cell lines CNE-2 and HONE-1 were originally obtained from NPC patients and maintained in our laboratory in RPMI-1640 (Gibco/BRL, Gaithersburg, MD) supplemented with 10% heat-inactivated fetal bovine serum (Thermo Scientific HyClone, Logan, UT). Cells were incubated in a humidified 5% CO_2 _atmosphere at 37°C. ApoG2, which was supplied by Dajun Yang (*Ascenta Therapeutics Incorporation, Malvern, Pennsylvania*), was dissolved in pure dimethyl sulfoxide (DMSO) at the stock concentration of 20 mmol/l and stored at -20°C. 3-(4,5 dimethylthiazol-2-yl)-2, 5-diphenyltetrazolium (MTT) were purchased from Sigma-Aldrich (St. Louis, MO). In *in vivo *experiments, for intraperitoneal (i.p.) injection, ApoG2 was suspended in 0.5% sodium carboxymethylcellulose and prepared on the day of use.

### MTT Assay

NPC cell viability was assessed using an MTT assay based on mitochondrial conversion of MTT from soluble tetrazolium salt to an insoluble colored formazan precipitate, which was dissolved in DMSO and quantitated using a spectrophotometer (Thermo Multiskan MK3; Thermo Fisher Scientific, Waltham. MA) with optical density (OD) values [12]. NPC cells were plated in 96-well culture clusters (Costar, Cambridge, MA) at a density of 15,000 to 25,000 cells/ml. Serial dilutions of ApoG2 were prepared from a stock solution to the desired concentrations. The final DMSO concentration was less than 0.1% (v/v). All experimental concentrations of ApoG2 were prepared in triplicate. Cells were treated with ApoG2 for 24, 48 and 72 h. Before termination of treatment, cells were incubated with 10 μl of 10 mg/ml MTT for 4 h. Then MTT and medium were depleted, and 100 μl of DMSO was added to the plates. The percent absorbance of Apog2-treated cells relative to the control (DMSO treated cells, DMSO concentration was less than 0.1%) was plotted as a linear function of the drug concentration. The antiproliferative effect of ApoG2 on NPC cells was measured as the percent of viable cells relative to the control using the equation 100% × OD_T_/OD_C_, in which OD_T _is the mean OD value of the ApoG2-treated treated samples and OD_C _is the mean OD value of the control samples. The 50% inhibitory concentration of ApoG2 was defined as the concentration of the drug required to achieve 50% growth inhibition relative to control populations.

### Cell Cycle Analysis

Untreated control and ApoG2-treated CNE-2 cells were harvested, washed twice with phosphate-buffered saline (PBS), and fixed dropwise with 2 ml of 70% ice-cold ethanol. After cells fixed overnight at 4°C, cells were then washed twice with PBS; cells were then incubated in RNase (20 μg/ml) at 37°C for 30 min to avoid staining the RNA. Next, the cells were washed once with PBS; PI was added to samples at a final concentration of 15 μmol/l, and after 5 min of incubation, the cells were analyzed using flow cytometry (Beckman Coulter, Fullerton, CA). The percentages of the nuclei in CNE-2 cells at each phase of the cell cycle (G1, S, G2/M) were calculated using the MultiCycle software program (Phoenix Flow Systems, San Diego, CA).

### Immunoblot Analysis

Protein analysis using immunoblotting and immunoprecipitation was performed with primary antibodies against p53 (sc-126; Santa Cruz Biotechnology, Santa Cruz, CA), p21 (sc-6246; Santa Cruz Biotechnology), c-Myc (sc-42; Santa Cruz Biotechnology), cyclin E (sc-481; Santa Cruz Biotechnology), cyclin D1 (sc-8396; Santa Cruz Biotechnology), and actin (clone AC-15; Sigma-Aldrich) as described previously [13]. Total cell lysates were harvested, electrophoresed using 12% sodium dodecyl sulfate-polyacrylamide gel electrophoresis, and transferred to polyvinylidene difluoride membranes (Roche, Grenzacherstrasse, Basel, Switzerland). Immunoblotting was performed using the primary antibodies described above followed by detection of protein expression using secondary antibodies conjugated with horseradish peroxidase (Cell Signaling Technology, Danvers, MA), and blots were developed using ECL chemiluminescent reagent (Cell Signaling Technology).

### RNA Interference

Transient small interfering RNA (siRNA) transfection was performed using Lipofectamine 2000 (Invitrogen, San Diego, CA) and 50 nM siRNA oligonucleotides. Commercially purchased siRNAs (Ribobio, Guangzhou, People's Republic of China) were scrambled (nontargeting), glyceraldehyde-3-phosphate dehydrogenase siRNA, and c-Myc siRNA. The three independent oligonucleotides designed for the c-Myc siRNA sequences were 5'-CAGAAATGTCCTGAGCAAT-3', 5'-AAGGTCAGAGTCTGGATCACC-3', and 5'-AAGGACTATCCTGCTGCCAAG-3'. The siRNA duplexes were introduced into CNE-2 cells according to the siRNA manufacturer's protocol. After transfection with siRNA for 48 h, cells were harvested for immunoblots and cell cycle analysis. The scrambled siRNA construct was used as a negative control.

### In vivo treatment and immunohistochemistry assay

Four-week-old athymic nude (nu/nu) mice obtained from the Animal Center of Southern Medical University (Guangdong, China) received subcutaneous injection of 1 × 10^7 ^CNE-2 cells in each axillary area. When subcutaneous tumors developed to more than 1,500 mg, mice were euthanized and tumors were dissected and mechanically dissociated into equal pieces to be transplanted into the flank areas of a new group of mice. When xenograft tumors became palpable (about 0.1 mm^3^), mice were randomly divided into control (0.5% sodium carboxymethylcellulose solution) and ApoG2 (120 mg/kg of body weight given by intraperitoneal injection daily) groups. Each group contained 8 mice, and there was no difference in tumor size between groups. Based on our lab's policy, when xenograft tumors developed to more than 1,000 mg, mice were euthanized and tumors were dissected and weighed. Immunohistochemical analysis was performed on tissue-sample sections of CNE-2 xenografts obtained from control and ApoG2. All samples were stained with hematoxylin and eosin and microscopically examined to confirm the CNE-2 cell origin. Sections were then stained with c-Myc (#; Santa Cruz) at 4°C overnight and then visualized using diaminobenzidine (DAB) (DAKO Liquid DAB, Dako, Carpinteria, CA) as peroxidase substrates.

### Statistical analysis

All analyses to compare the significance of measured levels were completed using the unpaired *t*-test by SPSS 16.0 software.

## Results

### ApoG2 Inhibits Cell Proliferation of NPC cells

**Figure 1:**
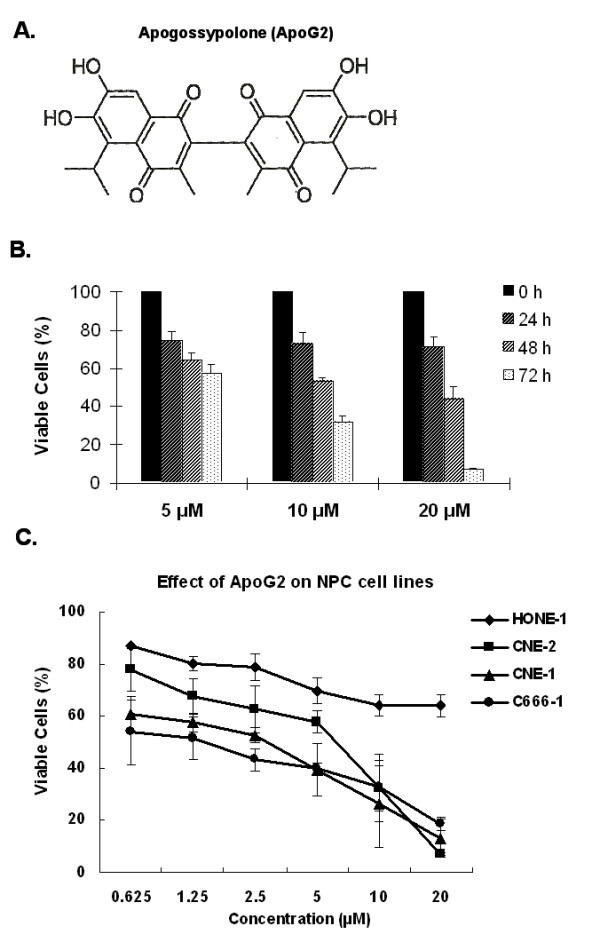
**ApoG2 and its inhibitory effect on CNE-2 cell proliferation**. **(A) **The chemical structure of ApoG2. **(B) **Effect of ApoG2 on NPC cell survival. Cells were exposed to 5, 10, and 20 *μ*M ApoG2 for 24, 48, and 72 h. Compared to control cells (treated with 0.1% DMSO), percentage of viable cells in treated samples was measured using an MTT assay (mean ± standard deviation for three experiments). **(C) **The inhibitory effect of ApoG2 on four NPC cell lines (HONE-1, CNE-2, CNE-1 and C666-1) was compared after 72-hr treatment. Points, average of three experiments; bars, SD.

Our previous work demonstrated that ApoG2 (Fig. 1A, the chemical structure of ApoG2) could significantly kill NPC cells and suppress the growth of NPC xenografts in nude mice. In this study, we reevaluated the antiproliferative effect of ApoG2 on CNE-2 cells using an MTT assay. We treated CNE-2 cells with 5, 10 and 20 μM ApoG2 for 24, 48 and 72 h. This treatment resulted in dose- and time-dependent inhibition of cell proliferation (Fig. 1B). At 10 and 20 μM, ApoG2 inhibited about 60% and 90% of the cell growth, respectively, at 72 h.

Moreover, among four NPC cell lines C666-1 (EBV infected), CNE-1 (highly differentiated), CNE-2 (poorly differentiated) and HONE-1 (poorly differentiated), ApoG2 treatment resulted in a tremendous inhibition of cell proliferation in C666-1, CNE-1 and CNE-2 NPC cell lines. At 10 μM, ApoG2 inhibited more than 60% of the cell growth of C666-1, CNE-1 and CNE-2 cells at 72 h. In contrast, only about 30% of HONE-1 cell proliferation was inhibited by 10 μM ApoG2 treatment for 72 h.

### AapoG2 Treatment Induces NPC Cells Arresting in S Phase of Cell Cycle

**Figure 2:**
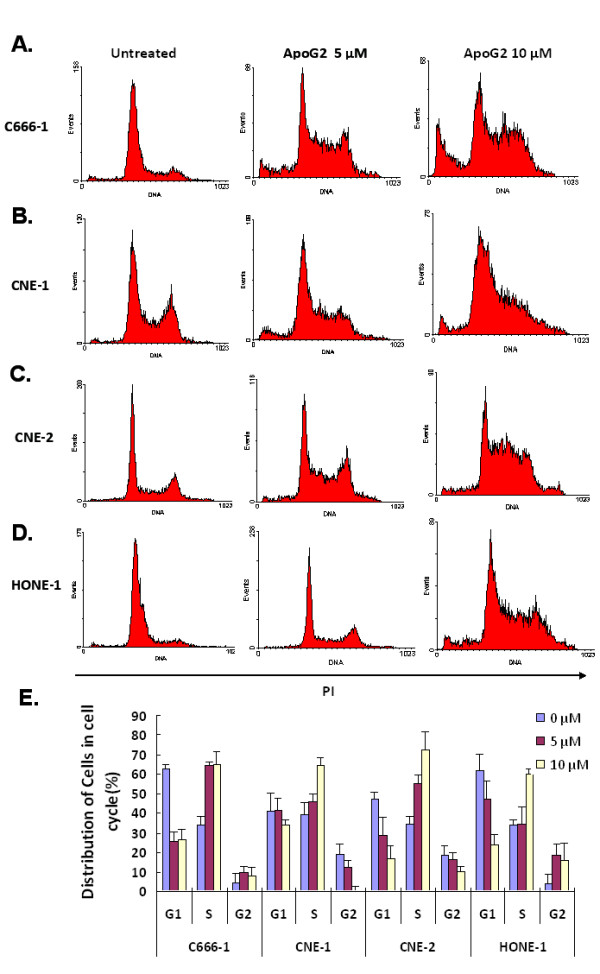
**Arrest of NPC cells at S phase of the cell cycle by treatment with ApoG2**. Arrest of NPC cells at S phase by ApoG2. C666-1 **(A)**, CNE-1 **(B)**, CNE-2 **(C) **and HONE-1 **(D) **cells were treated with 5 and 10 *μ*M ApoG2 for 48 h. DNA cell cycle analysis was performed using PI staining and flow cytometry. Each histogram is representative of three experiments. **(E) **Cell cycle analysis showed that ApoG2 treatment induced a conspicuous increasing of cells in S phase in four NPC cell line at 48 h. Bar heights, average of three independent experiments; bars, SD.

Gossypol reportedly induces cell cycle arrest in prostate cancer cells and colon cancer cells [8,9]. To determine whether ApoG2 could also induce cell cycle arrest in NPC cells, we performed a cell cycle analysis using flow cytometry. The results showed the same with our previous work [5] that, at 48 h after treatment, ApoG2 did not induce obvious cell apoptosis in NPC cells and little cells were accumulated in sub-G1 phase. Instead, ApoG2 induced cell cycle arrest at the DNA synthesis (S) phase in a large percentage of NPC cells at this time. More than 60% of C666-1, CNE-1 and CNE-2 cells were arrested at S phase at 48 h after exposure to 5 and 10 μM ApoG2, whereas only 34%, 39% and 35%, respectively, of untreated C666-1, CNE-1 and CNE-2 cells were arrested at S phase (Fig. 2A–C).

Because we observed that another NPC cell line, HONE-1, was much less sensitive to ApoG2 treatment and exhibited a much higher 50% inhibitory concentration value of ApoG2 (more than 10-fold) than C666-1, CNE-1 and CNE-2 cells (data not shown), we assessed the effect of ApoG2 on the cell cycle in this cell line. Treatment with 10 μM ApoG2 induced about 60% HONE-1 cells arresting at S phase (Fig. 2D); in comparison, only 34% of untreated HONE-1 cells were arrested at S phase of the cell cycle. These data implied that ApoG2-induced cell cycle arrest is not correlated with the sensitivity of cells to ApoG2, because in both ApoG2-sensitive NPC cells and ApoG2-insensitive HONE-1 cells, ApoG2 treatment could result in significant cell cycle arrest. These data also implied that ApoG2-induced cell cycle arrest was not caused the inhibition of Bcl-2 proteins and other molecular mechanisms might be involved in ApoG2-induced cell cycle arrest in NPC cells.

### Downregulation of c-Myc Expression Leads to Cell Cycle Arrest by ApoG2 in NPC cells

**Figure 3:**
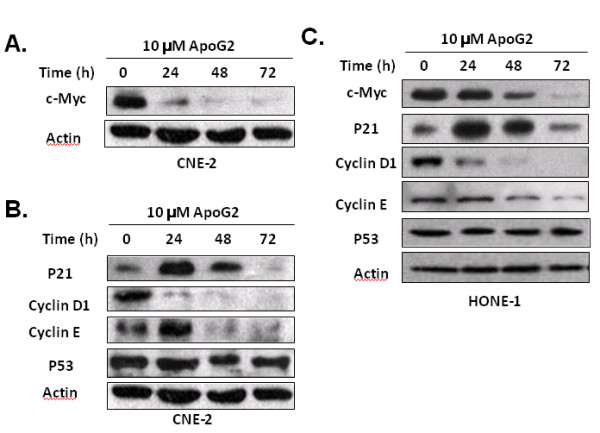
**Treatment with ApoG2 induces alterations in the expression of c-Myc, p21, and cyclins**. **(A) **The effect of ApoG2 on the expression of c-Myc. CNE-2 cells were incubated with 10 *μ*M ApoG2 for 24 to 72 h, and cell lysates were analyzed using immunoblotting. **(B) **The effect of ApoG2 on the expression of molecules downstream from c-Myc. After treatment with ApoG2, CNE-2 cell lysates were analyzed using immunoblotting with anti-p21, -cyclin D1, -cyclin E, and -p53 antibodies. **(C) **The effect of ApoG2 on cell cycle-regulatory molecules in HONE-1 cells. Cells were treated with 10 *μ*M ApoG2 for 24 to 72 h, and cell lysates were analyzed using immunoblotting.

Because researchers have reported that cell cycle-regulating molecules, such as p21, p53, and TGF-β1, play roles in gossypol-induced cell cycle arrest [9,14], we hypothesized that ApoG2 can also modify some cell cycle regulators, resulting in cell cycle arrest in NPC cells. Consistent with our hypothesis, treatment with 10 μM ApoG2 significantly decreased the level of c-Myc protein expression at 24 h in CNE-2 cells (Fig. 3A). Moreover, expression of p21 protein was upregulated as early as 24 h and gradually returned to low level at 72 h since most of the CNE-2 cells were dead at this time (Fig. 3B); unlike p21, expression of both cyclin D1 and cyclin E were downregulated following the degradation of c-Myc. We observed no changes in p53 protein expression (Fig. 3B). Similar changes in the c-Myc pathway were also detected in ApoG2-treated HONE-1 cells (Fig. 3C), which was in agreement with the results of cell cycle analysis that ApoG2 induced cell cycle arrest in both sensitive CNE-2 cells and insensitive HONE-1 cells.

### Downregulation of c-Myc Expression by siRNA Leads to Cell Cycle Arrest at S Phase in CNE-2 Cells

**Figure 4:**
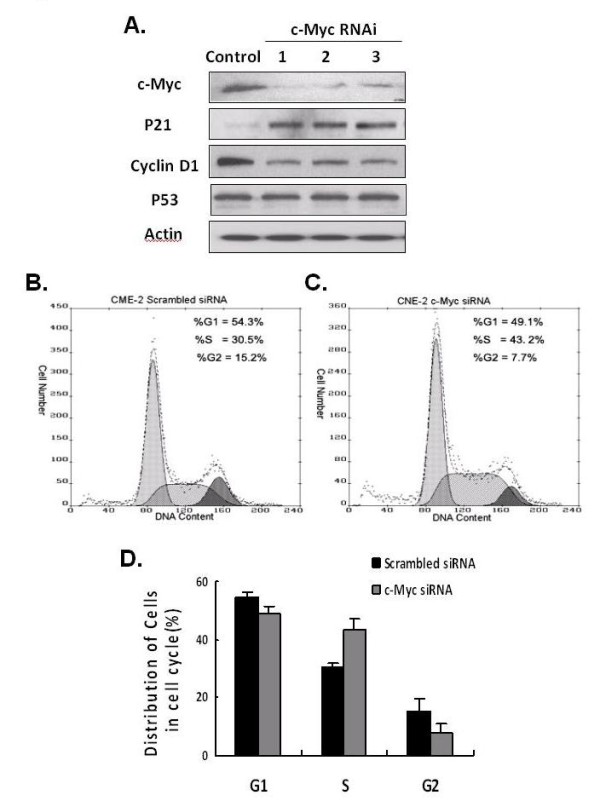
**The effect c-Myc siRNA transfection on c-Myc downstream molecules and cell cycle distribution**. **(A) **Comparison of the effect of c-Myc siRNA and scrambled (nontargeting) siRNA on the expression of c-Myc downstream molecules. Transfection of CNE-2 cells with c-Myc or scrambled (nontargeting) siRNA for 48 h. Cells were then subjected to Western blotting using anti-c-Myc, -cyclin D1, -p21, and -p53 antibodies as described in Materials and Methods. Comparison of the effect of scrambled (nontargeting) siRNA **(B) **and c-Myc siRNA **(C) **on cell cycle distribution of CNE-2 cells using PI staining and flow cytometry. Each histogram is representative of three experiments. **(D) **Analysis of cell cycle distributions showed that, compared to srambled siRNA, c-Myc siRNA induced a conspicuous increasing of cells in S phase in CNE-2 cells at 48 h. Bar heights, average of three independent experiments; bars, SD.

Authors have reported that the oncoprotein c-Myc regulates the expression of p21 and cyclins, increases cyclin D-CDK4 activity, and facilitates cell cycle progression [15]. Also, Fan et al. found that upregulated expression of c-Myc protein in NPC cells contributed to unrestricted cell proliferation, metastasis, and tumor progression [16]. In our study, the immunoblots data indicated that suppression of the c-Myc pathway might be responsible for ApoG2-induced cell cycle arrest in NPC cells. To test this hypothesis, we used three siRNA oligonucleotides (Ribobio, Guangzhou, China) to knock down c-Myc protein in CNE-2 cells. As shown in fig. 4A, all these three oligonucleotides significantly suppressed the expression of c-Myc protein; the reduction in c-Myc expression led to upregulation of p21 expression and downregulation of cyclin D expression. Cell cycle analysis showed that incubation with scrambled siRNA resulted in a significantly lower CNE-2 cell population arrested at S phase than did incubation with c-Myc siRNA (Fig. 4B and 4C). Compared to srambled siRNA, c-Myc siRNAs induced conspicuous increasing of cells in S phase in CNE-2 cells at 48 h (Fig. 4D). Based on these results, we suggested that suppression of the c-Myc pathway by ApoG2 leads directly to cell cycle arrest in NPC cells.

**Figure 5:**
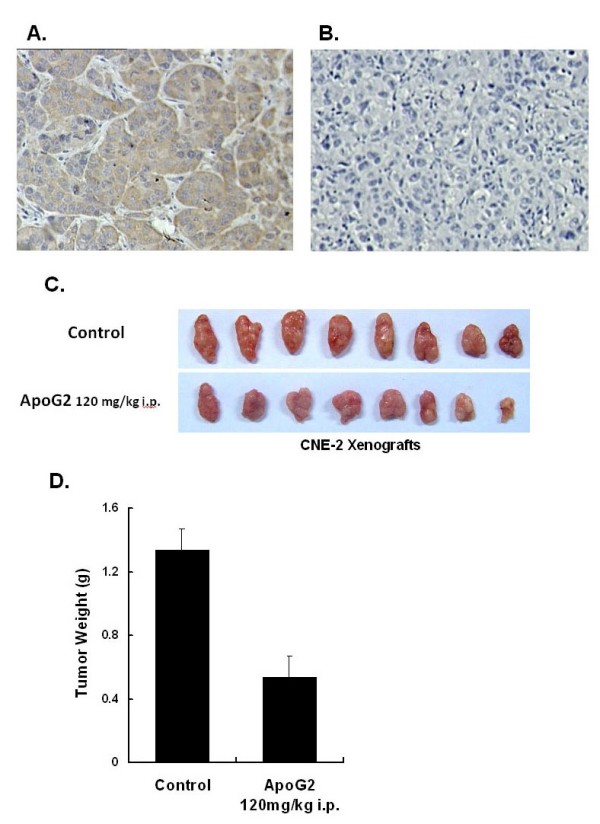
**Analysis of the impact of ApoG2 *in vivo *on c-Myc expression and tumor growth in CNE-2 xenografts**. The tumor tissues from ApoG2 (120 mg/kg intraperitoneal injection daily) treatment were obtained at the end of 12 days of treatment. Immunochemistry analysis of c-Myc expression in CNE-2 xenograft tumor sections after NS treatment **(A) **or ApoG2 treatment **(B)**, magnification, × 80. **(C) **Photographs of CNE-2 xenografts from NS treatment and ApoG2 treatment groups. When all tumors of the control group exceeded 1 g in weight, the animal experiment was terminated and mice were killed. The tumors were taken out for weighing and comparing the effect of ApoG2 on tumors. **(D) **Antitumor acitivity of ApoG2 in CNE-2 xenograft-bearing nude mice. Compared to control (NS treatment) mice, ApoG2 treatment greatly suppressed tumor weight. Bar heights, average weight of eight CNE-2 xenograft tumors; bars, SD.

ApoG2 inhibites c-Myc expression level in CNE-2 xenografts in nude miceTo assess the effect of ApoG2 on c-Myc expression *in vivo*, we used the CNE-2 xenografts nude mice model. When control xenografts developed to more than 1,000 mg, all mice were euthanized and tumors were dissected, weighed and fixed for immunochemistry assay. As shown in fig. 5A and 5B, compared to NS (normal saline) treatment group, ApoG2 treatment provoked a significant reduction in c-Myc expression level in CNE-2 xenografts. Antitumor activities of ApoG2 (120 mg/kg i.p. injection once every three days) against CNE-2-bearing nude mice was measured by weighing the weight of CNE-2 xenografts (Fig. 5C). As shown in fig. 5D, compared to control treatment, ApoG2 could significantly inhibit tumor weight in CNE-2 xenografts (p < 0.001).

## Discussion

ApoG2 is the oxidation product of gossypol and has two aromatic hydrocarbon quinone groups. Authors have reported that aromatic hydrocarbon quinone stimulates ROS production in hepatic cells [17]. As we known, elevated ROS levels may damage cellular DNA, inducing generation of oxidized bases, DNA strand breaks, and stop of DNA replication, in ApoG2-treated CNE-2 cells. Recent studies provided evidence that multiple chemopreventive agents can cause generation of ROS to trigger signal transduction, culminating in cell cycle arrest and/or apoptosis [18,19]. However, Van Poznak et al. and Zhang et al. suggested that gossypol-induced cell cycle arrest is associated with alterations of p21, cyclin D1, and p53 and showed that p21 is the first target of gossypol to inhibit cell growth *in vivo *[9,20]. Our data indicated that ApoG2 induced massive cells arrest at S phase of the cell cycle not only in ApoG2-sensitive NPC cells but also in ApoG2-insensitive HONE-1 cells (Fig. 3). Results of signaling pathway analysis showed that downregulation of c-Myc protein expression was the major upstream event in ApoG2-induced cell cycle arrest in NPC cells (Fig. 4). Basically, the effect of c-Myc on cell cycle is to drive quiescent cells into the cell cycle, and shortening G1 and promoting S phase entry thereby. The down-regulation of c-Myc should cause a preferential G1/S arrest rather than S arrest. However, in NPC cells, although p53 was highly expressed and its expression was never downregulated by ApoG2 in this study, p53 was mutated and functionally impaired by Epstein-Barr virus nuclear antigen 5 and deltaN-p63 in NPC cells [21,22]. In this scenario of malfunction of G1-S checkpoint p53, c-Myc was a main factor accounting for ApoG2-induced S phase arrest. P21 and cyclins were followed by downregulation of c-Myc expression.

c-Myc is not only a central regulator of cell proliferation but also induces cells to undergo apoptosis, unless specific signals provided by oncogenes block the apoptosis pathway [23]. Notably, NPC cells consistently harbor EBV DNA and express EBV proteins, LMP1 and BARF1; these proteins stimulate oncogenic antiapoptotic Bcl-2 proteins to protect host cancer cells from apoptosis [24-27]. ApoG2 is a potent inhibitor of antiapoptotic Bcl-2 proteins and its treatment could remove the protective effect of Bcl-2 proteins and facilitate apoptosis. In this case, downregulation of c-Myc expression by ApoG2 on one hand could let cells away from c-Myc-induced apoptosis and on other hand led to cell cycle arrest. However, by inhibiting Bcl-2 proteins, ApoG2 still helped release pro-apoptotic proteins, such as Bax and Bak, and irreversibly damaged mitochondria and induced cell apoptotic [5].

Gossypol is clinically used in China to treat adenomyosis and hysteromyoma because of its ability to inhibit estrogen and progesterone by competitively binding to the estrogen receptor and progesterone receptor [28]. c-Myc is a well-established target of estrogen action and plays a role in controlling cell cycle progression. Anti-estrogen treatment is reported to be able to cause an acute decrease in c-Myc expression, a subsequent decline in cyclin D1 expression, and, ultimately, inhibition of DNA synthesis and arrest of cells in a quiescent state [29]. Estrogen receptor and progesterone receptor are known to be highly expressed in NPC cells, and their expression is considered a sign of distant metastasis and a poor prognosis [30]. Based on our findings, we suggest that ApoG2-induced cell cycle arrest is dependent on ApoG2's downregulation of c-Myc expression. Use of ApoG2 to treat NPC may suppress the activity of estrogen and progesterone and reduce the incidence of distant metastasis and local relapse.

The concept of targeted biological therapy for cancer has emerged over the past decade. Clinical trials studying the efficacy and tolerability of these targeted agents has shown that most tumors depend on more than one signaling pathway for their growth and survival. Therefore, investigators pursue different strategies to inhibit multiple signaling pathways by developing multitargeted agents [31]. The recent U.S. Food and Drug Administration approval of sorafenib and sunitinib, which target vascular endothelial growth factor receptor, platelet-derived growth factor receptor, FLT-3, and c-Kit, marks the use of a new generation of multitarget anticancer drugs [32]. Our study show that ApoG2 is one such multitarget agent that targets both the antiapoptotic and cell cycle progression pathway in NPC cells by blocking antiapoptotic Bcl-2 proteins and the c-Myc oncogenic pathway. These findings provide an entirely new concept for the use of ApoG2 in cancer therapy.

## Conclusion

Our findings indicated that ApoG2 can potently disturb the proliferation of NPC cells by suppressed c-Myc signaling pathway. This data suggested that the inhibitory effect of ApoG2 on NPC cell cycle proliferation might contribute to its use in anticancer therapy.

## Abbreviations

ApoG2: apogossypolone; DMSO: dimethyl sulfoxide; EBV: Epstein-Barr virus; LMP1: latent membrane protein 1; MTT: (3-[4,5-dimethylthiazol-2-thiazolyl]-2,5-diphenyltetrazolium bromide; NPC: nasopharyngeal carcinoma; OD: optical density; PBS: phosphate-buffered saline; Rb: retinoblastoma gene; siRNA: small interfering RNA; TGF-β1: transforming growth factor-β1.

## Competing interests

The authors declare that they have no competing interests.

## Authors' contributions

YXZ was responsible for study design. DY and XFZ performed the experiments and drafted the manuscript. JS participated in the data analysis and western-blot. All authors read and approved the final manuscript.
